# Serial measurement of lipid profile and inflammatory markers in patients with acute myocardial infarction

**DOI:** 10.17179/excli2014-671

**Published:** 2015-04-10

**Authors:** Amit Kumar Shrivastava, Harsh Vardhan Singh, Arun Raizada, Sanjeev Kumar Singh

**Affiliations:** 1 Department of Biochemistry, Sudha Rustagi College of Dental Sciences & Research, Faridabad, India; 2 Department of Biochemistry, North Delhi Municipal Corporation Medical College & Hindu Rao Hospital, Delhi, India; 3 Department of Pathology and Laboratory Medicine, Medanta-The Medicity, Gurgaon, India; 4 Department of Biochemistry, G. R. Medical College, Gwalior, India

**Keywords:** Acute myocardial infarction, lipid profile, inflammatory markers, serial measurement

## Abstract

Serum concentration of lipids and lipoproteins changes during the course of acute coronary syndrome as a consequence of the inflammatory response. The objective of this study was to evaluate the effect of acute myocardial infarction (AMI) on the levels of lipid profile and inflammatory markers. We investigated 400 patients with AMI who were admitted within 24 h of onset of symptoms. Serum levels of total cholesterol (TC), triglyceride (TG), low density lipoprotein (LDL) and high density lipoprotein (HDL) were determined by standard enzymatic methods along with high sensitive C-reactive protein (hs-CRP) (latex enhanced immunoturbidimetric assay) and cytokines, interleukin (IL)-6 and IL-10 (quantitative ''sandwich'' enzyme-linked immunosorbent assay). The results indicate a trend of reduced TC, LDL, and HDL, and elevated TG levels, along with pro- and anti-inflammatory markers (p < 0.001), between day 1 and the day 2 serum samples of AMI patients. However, corrections in the serum levels have been observed at day 7. Our results demonstrate significant variations in the mean lipid levels and inflammatory markers between days 1, 2 and 7 after AMI. Therefore, it is recommended that the serum lipids should be assessed within 24 hours after infarction. Early treatment of hyperlipidemia provides potential benefits. Exact knowledge regarding baseline serum lipids and lipoprotein levels as well as their varying characteristics can provide a rational basis for clinical decisions about lipid lowering therapy.

## Introduction

For many years, the proclamation that coronary heart disease (CHD) was not attributable to traditional risk factors in up to 50 % of cases was finally submerged after careful and detailed re-analysis (Miller, 2008[18]). Dyslipidemia is still a major risk factor for CHD. Epidemiological studies have conclusively linked high levels of total cholesterol (TC) and low-density lipoprotein cholesterol (LDL-C) and low levels of high-density lipoprotein cholesterol (HDL-C) with CHD incidence and mortality (Yokokawa et al., 2011[35]). It is well known that early treatment of hyperlipidemia following acute myocardial infarction (AMI) provides potential benefits and reduces the morbidity and mortality of CHD. However, the levels of lipid and lipoproteins change during acute illnesses that cause delay in treatment choice (Balci, 2011[3]). During tissue necrosis, acute phasic changes occur that alter the lipid profile levels post acute coronary events. Modifications of serum lipids after AMI include reductions in TC, LDL and HDL, in the range of 10 - 20 %, with reciprocal increases in triglyceride (TG) approximating 20 - 30 % (Miller, 2008[18]). Several mechanisms accounting for these changes include the acute phase response associated with up-regulation of LDL-receptor (R) activity and reduction in several pivotal HDL regulatory proteins. From many clinical studies it is clear that phasic changes do occur in patients following AMI and therefore there is a recommendation for detection of hyperlipidemia in patients with AMI that the serum lipids should be assessed either within 24 hours after infarction or after 2-3 months of AMI (Nigam, 2007[20]). Accurate knowledge of baseline lipid levels may affect the initiation of lipid-lowering therapy, the selection of a specific statin and its dosage, and recognition of the potential need for adjunctive lipid therapy, and may influence the patient's willingness to adhere to a recommendation for long-term lipid-lowering therapy (Pitt et al., 2008[22]).

Besides alterations in the lipoproteins, acute-phase response is also associated with changes in serum concentration of inflammatory markers. There is an intra-cardiac inflammatory response in AMI that appears to be the result of the evolution of myocardial necrosis, as shown by higher C-reactive protein (CRP) and interleukin (IL)-6 levels in patients with major adverse cardiac events (Raposeiras Roubin et al., 2013[23]). Several population based prospective studies of CHD have reported a close association of subtle, prolonged increases in baseline high sensitive (hs)-CRP levels with cardiovascular risk (Casas et al., 2008[7]). The majority of authors concur in that the admission hs-CRP concentration reflects the baseline inflammatory status of the patient; thus, patients with AMI and high hs-CRP levels at admission usually experience more cardiovascular complications during follow-up (Bursi et al., 2007[6]). IL-6 is a multifunctional cytokine regulating humoral and cellular responses and playing a central role in inflammation and tissue injury. Similarly to CRP, whose synthesis is stimulated by IL-6, high circulating concentrations of IL-6 are associated with increased risk of cardiovascular events (Swerdlow et al., 2012[27]). On the other hand, IL-10 is a centrally operating anti-inflammatory cytokine that plays a crucial role in the regulation of the innate immune system and can suppress the production of a variety of proinflammatory molecules (Biswas et al., 2010[5]). The expression of IL-10 has been demonstrated in both coronary arteries and atherosclerotic plaque. Furthermore, serum levels of IL-10 have been shown to be greater in individuals with atherosclerosis compared to controls, suggesting IL-10, as an anti-inflammatory molecule, may be elevated in response to the pro-inflammatory environment of atherosclerosis (Lakoski et al., 2008[16]).

The main aim of the present study was to examine the changes in serum lipid profile in AMI patients in different time intervals. To analyze the association of lipid profile with inflammation followed by acute coronary events, we also estimated the levels of inflammatory markers hs-CRP and IL-6 along with anti-inflammatory marker IL-10.

## Material and Methods

A total of 520 patients with suspected AMI consecutively admitted to emergency department in which 400 patients with confirmed AMI were recruited from the coronary care unit and cardiology department of a tertiary care hospital in Gurgaon, India. We included patients if they met all the following criteria: (a) all patients had AMI at baseline; (b) blood sample for lipid profile and inflammatory markers estimation was obtained within 24 h from the onset of symptoms. Individuals with rheumatic disease, chronic liver diseases, renal disorders, cancer, sepsis and patient critically ill with less than one month duration infectious diseases and surgical procedure in 3 month duration, AMI and stroke within the past six months, severe congestive heart failure or cardiogenic shock, regular or chronic use of anti-inflammatory drugs in the previous two months were excluded from the study.

Clinical history and physical examination data, focusing on characteristics of chest pain and presence of cardiovascular risk factors were recorded for every patient. Serial electrocardiograms and cardiac enzymes were also obtained in all patients. MI was defined by detection of rise in cardiac biomarkers of necrosis (cTroponin I) with at least 1 value above the 99^th^ percentile upper reference limit, together with evidence of myocardial ischemia with at least 1 of the following: electrocardiographic changes indicative of new ischemia (new ST-T changes or new left bundle branch block), new pathological Q waves in at least 2 contiguous leads, imaging evidence of new loss of viable myocardium, or new wall motion abnormality (Thygesen et al., 2007[30]). Diabetes was defined as a previous diagnosis, use of anti-diabetic medicines, or a fasting venous blood glucose level ≥ 126 mg/dL. Hypertension was defined as patient systolic blood pressure > 140 mmHg and/or diastolic blood pressure > 90 mmHg at rest, over a series of repeated measurements, or on treatment with antihypertensive medications. Body mass index (BMI) was calculated as weight (kg) divided by the square of height (m^2^) and a patient with a value above 30 was categorized as obese. Level of TC more than 200 mg/dL was used for the identification of hypercholesterolemia. 150 controls were also selected from blood donor, hospital staff and from the health check up individuals who met the matching criteria of age, sex, and smoking status. These healthy controls were screened for diabetes, hypertension and dyslipidemia. Subjects were informed about the study in detail and written consent of patients was also taken before starting the study. All ethical measures were taken prior to start the study.

Serum lipid profile along with inflammatory markers was measured only on the fasting blood samples within the first 24 h of the onset of symptoms of MI and again at day 2 and day 7 post-MI. Serum TC, TG, HDL-C and LDL-C levels were measured by an enzymatic colorimetric method using reagents of VITROS chemistry products on automated clinical chemistry analyzers. Serum levels of hs-CRP were determined by latex enhanced immunoturbidimetric assay with the use of reagents and calibrators from Roche diagnostics. The levels of IL-6 and IL-10 were estimated by means of commercially available quantitative “sandwich” enzyme-linked immunosorbent assay (ELISA) kits obtained from R&D Systems, according to the instructions of the manufacturer.

Statistical Package for the Social Sciences 21 (SPSS 21) was used for all statistical analyses. All the descriptive variables were expressed as the mean ± standard deviation (SD). Independent sample t-tests were used to compare the mean values of variables between the AMI and control groups, whereas chi-square test was used for association between two categorical variables. To evaluate the association between serum samples of day 1, day 2 and day 7, the mean values were compared by one way analysis of variance (ANOVA) followed by Tukey's post hoc test. A probability value p < 0.05 was considered statistically significant.

## Results

Table 1(Tab. 1) shows the baseline clinical and laboratory characteristics of study participants. The mean age of 400 patients with AMI was 59.07 ± 7.34 years and the patient group was comprised of 155 females and 245 males, whereas, mean age of 150 healthy controls was 59.48 ± 7.73 years and it was comprised of 57 females and 93 males (both the groups have a same sex distribution ratio). The proportion of hypertension was 42 % and diabetes mellitus was 25 % for AMI patients. 140 and 85 AMI patients had already diagnosed hypertension and diabetes, respectively, whereas 32 (hypertension) and 13 (diabetes) patients were diagnosed during their investigations in the hospital. There were no significant differences between AMI patients and controls in terms of age, gender, diabetes, hypertension, smoking and drinking habits. 

Table 2(Tab. 2) shows mean values and SD of all parameters studied in both groups with test of significance using SPSS 21 statistical software. Baseline serum levels of TC (174.81 ± 24.14 vs. 169.35 ± 16.34 mg/dL, p = 0.003), TG (157.51 ± 45.67 vs. 149.65 ± 27.18 mg/dL, p = 0.014) and LDL-C (111.35 ± 25.81 vs. 105.55 ± 30.39 mg/dL, p = 0.039) were significantly higher in AMI patients as compared to the controls, whereas HDL-C was significantly higher (43.14 ± 11.34 vs. 49.21 ± 9.14 mg/dL, p < 0.001) in the latter group. Inflammatory markers hs-CRP (9.82 ± 5.63 vs. 1.03 ± 0.67 mg/L), and IL-6 (50.80 ± 25.12 vs. 14.81 ± 6.65 pg/mL) were found to be significantly higher (p < 0.001) and IL-10 (11.65 ± 8.95 vs. 13.92 ± 6.06 pg/mL, p = 0.002) were significantly lower in AMI patients than controls.

**Figure 1:**
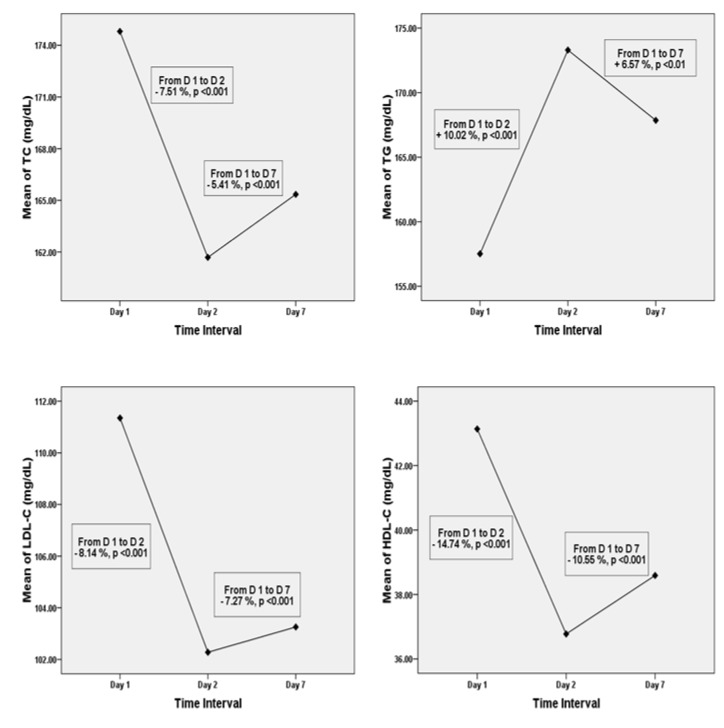
Mean levels of serum TC, TG, LDL-C, and HDL-C on Days 1, 2, and 7. TC; total cholesterol, TG; triglyceride, LDL-C; low density lipoprotein cholesterol, HDL-C; high density lipoprotein cholesterol, D; day

**Figure 2:**
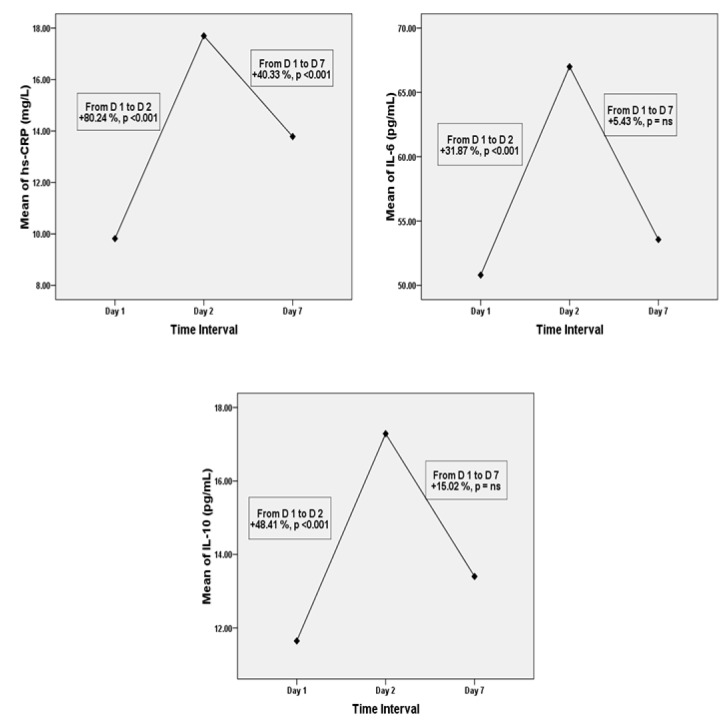
Mean levels of serum hs-CRP, IL-6 and IL-10 on days 1, 2, and 7. hs-CRP; high sensitive C-reactive protein, IL; interleukin, D; day, ns; non-significant

In AMI patients, all serum lipid levels changed significantly between day 1 post-MI (i.e., within 24 h) - day 7 post-MI (Table 3(Tab. 3), Figure 1(Fig. 1)). From day 1 to day 2 post-MI, serum TC levels (174.81 ± 24.14 vs. 161.68 ± 30.77 mg/dL), LDL-C levels (111.35 ± 25.81 vs. 102.28 ± 23.23 mg/dL), and HDL-C levels (43.14 ± 11.34 vs. 36.78 ± 10.31 mg/dL) decreased significantly (p < 0.001). On the contrary, the serum TG levels increased significantly (p < 0.001) from 157.51 ± 45.67 on day 1 to 173.30 ± 48.79 mg/dL on day 2. Although, there were some improvements in lipid profile levels on day 7, they failed to reach the baseline levels. Laboratory findings demonstrated there were significant fluctuations in the levels of inflammatory markers of AMI patients from day 1 - day 7 (Figure 2(Fig. 2)). Serum levels of hs-CRP and IL-6 that were measured at day 2 (17.70 ± 8.49 & 66.99 ± 24.35) after AMI were significantly higher (p < 0.001) than at day 1 (9.82 ± 5.63 & 50.80 ± 25.12) and day 7 (13.78 ± 7.54 & 53.56 ± 25.77). On the other hand, anti-inflammatory cytokine IL-10 levels seemed to increase from day 1 to day 2 (11.65 ± 8.95 vs. 17.29 ± 13.54 pg/mL, p < 0.001) and to decrease again by day 7 (13.40 ± 10.97 pg/mL) (Table 3(Tab. 3)).

## Discussion

A series of changes in lipid metabolism occur during acute phase response. As a result, plasma TG level increases, while HDL, LDL and TC levels decrease, demonstrated by many studies (Wattanasuwan et al., 2001[32]). There is no consensus with respect to timing of lipid and lipoprotein measurements in terms of proximity to the baseline values, the magnitude of the changes and when these changes reach maximum and basal values. A reduction in the magnitude of these changes is seen over time. First time Biorck et al. (1957[4]) reported that serum cholesterol levels decreased during MI. Since then, a wide range of changes in the serum lipid and lipoproteins following acute coronary events have been reported. In the present study, we found significant changes in TC levels throughout the study period in AMI patients. There are several reports indicating that cholesterol reduction takes place in the initial phase of an acute coronary event; thus, plasma levels determined at this point should be interpreted with caution. This reduction may be just a consequence of the inflammatory response, or it may be related to an increase in cellular uptake of cholesterol for tissue repair and hormonal synthesis (Correia et al., 2004[8]). A previous report by Khan et al. (2013[12]) showed significantly decreased level of TC in AMI patients.

Results of this analysis also suggest that directly measured serum LDL-C after admission for an AMI changes in a statistically way from day 1 to day 7. During acute phase reaction, LDL synthesis is increased. Despite that, LDL level decreases due to up-regulation of LDL-R activity (Balci, 2011[3]). Moreover, LDL particle size is smaller in patients with AMI as compared to non-AMI patients. In addition, the decrease in LDL-C concentration on day 2 of hospitalization may reflect causes related to hospitalization, such as altered oral intake or intravenous hydration (Pitt et al., 2008[22]). Ko et al. (2005[13]) in a large-scale review of patient records of admissions for MI also found decreased LDL-C between samples taken < 24 h (120 mg/ dL) and > 24 h (116 mg/dL) after admission. These results show agreement to the LATIN (Lipid Assessment Trial-Italian Network) study, where patients admitted within 12 h of symptom onset for MI or unstable angina found a mean 7 % (unstable angina) to 10 % (MI) decrease from admission to the next day in direct-measured LDL-C that persisted until discharge (Fresco et al., 2002[10]).

HDL-C levels, in our study, started falling from day 2 onwards. Similar results were reported by Nigam (2007[20]) and Kumar et al. (2009[15]) in previous Indian studies. Rosoklija et al. (2004[24]) concluded in their study that the optimal time for determining the HDL level were the first 24 hours of the actual event. In AMI, acute phase response has quantitative and qualitative effects on HDL and its contents. Inflammation decreases the level of HDL by increasing the activity of endothelial lipase and soluble phospholipase A2 and replacing the apolipoprotein-A1 in the HDL with serum amyloid A. Moreover, inflammation leads to changes in the size and function of the HDL (Ansell et al., 2005[2]). There is a decrease in the levels of several plasma proteins included in HDL-mediated reverse transport of cholesterol and inhibition of lipid oxidation during inflammation. Therefore, this remodeling creates functional alterations, including a decrease in cholesterol efflux capacity (Tsompanidi et al., 2010[31]). TG levels were also significantly changed during the study period in the patient group. 

In some previous studies Nigam (2007[20]) and Pitt et al. (2008[22]) also reported an increased level of TG after AMI. Hypertriglyceridemia is caused by increased lipoprotein production and decreased lipoprotein clearance. Increase in TG-rich lipoproteins is secondary to the re-esterification of plasma fatty acids. Clearance decreases mainly secondary to the inhibition of lipoprotein lipase activity (Navab et al., 2009[19]). Myocardial damage-induced stress increases the adrenergic-mediated lipolysis of the adipocytes, which leads to an increase in free fatty acids, TGs and lipoproteins. The mobilization of free fatty acids and hepatic secretion of very low density lipoprotein also increases the TG levels (Balci, 2011[3]). Based on these acute changes, the American College of Cardiology/American Heart Association (ACC/AHA) have supported a Class I recommendation for a fasting lipid profile analysis to be obtained within 24 h of admission for ACS (acute coronary syndrome) (Anderson et al., 2007[1]).

As expected, levels of hs-CRP and IL-6 were significantly higher in AMI patients as compared to the controls, and in patient's group levels were increased from day 1 to day 2, and then decreased from day 2 to day 7. In our patients, inflammatory markers level on day 2 were close to the peak of response, representing that the inflammatory process associated with myocardial necrosis was still ongoing and at its height at our second measurement. Similar results were shown by Yip et al. (2004[34]), Sheikh et al. (2012[25]) and Fan et al. (2011[9]). AMI is a multi-factorial disease, in which inflammatory processes play a central role (Biswas et al., 2010[5]). In this regard, CRP and IL-6 are considered to be the most important markers and have been extensively studied in recent years (He et al., 2004[11]; Tan et al., 2008[29]). CRP might not only mirror an inflammatory stimulus, but also have direct effects promoting atherosclerotic propagation and destabilizing plaque (Yip et al., 2004[34]). Our finding suggests that these substantially increased serum hs-CRP and IL-6 levels in the clinical setting of MI are due to the results of myocardial damage. Tissue necrosis is a potent acute-phase stimulus, and following MI, there is a major CRP response, the magnitude of which reflects the extent of myocardial necrosis (Pepys and Hirschfield, 2003[21]). Plasma CRP concentration increases following the cytokines activation in the initial hours of MI. CRP binds to phosphocholine group of necrotic myocardial cell membranes, facilitating complement activation, and thus promoting further inflammatory response, injury of myocardial cells, and expansion of necrosis (Swiatkiewicz et al., 2012[28]). In some recent studies, Khan et al. (2013[12]) and Raposeiras Roubin et al. (2013[23]) reported significantly increased levels of hs-CRP in AMI patients.

IL-6 is a pleiotropic cytokine with a broad range of humoral and cellular immune effects related to inflammation. Elevated IL-6 levels may contribute to the development and instability of atherosclerotic plaques by activation of leukocytes and endothelial cells or by the induction of various cytokines (Shinohara et al., 2012[26]). Furthermore, IL-6 decreases lipoprotein lipase (LPL) activity and monomeric LPL levels in plasma, which increases macrophage uptake of lipids (Fan et al., 2011[9]). In post-AMI patients, the activation of proinflammatory cytokines leads to high concentrations of inducible nitric oxide synthase, nitric oxide, and peroxynitrite, which have multiple harmful effects. In a recent study, Lopez-Cuenca et al. (2013[17]) reported that high IL-6 on day 1 to be associated with poor long-term outcomes in MI patients, which reaffirms the prognostic significance of the proinflammatory status during the initial phase of an ACS.

Consistent with some previous studies (Biswas et al., 2010[5]), we found significant low levels of IL-10 in patients with AMI as compared to controls at day 1 serum samples. Less clinical data is available regarding the role of anti-inflammatory cytokines in AMI. It recently has been demonstrated that IL-10 may act as a protective factor in atherosclerosis and suppresses the synthesis of proinflammatory cytokines (Krishnamurthy et al., 2009[14]). Moreover, IL-10 is expressed in both early and advanced human atherosclerotic plaques and inhibits many cellular processes including metalloproteinase production and tissue factor expression, which may play a role in the clinical expression of atherosclerotic plaque rupture or erosion (Lakoski et al., 2008[16]). Additionally, it may influence antigen presentation (including oxidized lipids) from macrophages and dendritic cells, and even stabilize rupture prone plaques by suppressing apoptotic pathways in foam cells (Welsh et al., 2011[33]). The mechanisms leading to increased release of IL-10 at day 2 in AMI patients remain unclear; however, it may be assumed that in severe inflammatory processes occurring in cardiovascular events, more IL-10 is produced as a compensatory phenomenon to inhibit continued pro-inflammatory cytokine production and inflammatory propagation, resulting in elevated levels of IL-10. Consistent with this, circulating IL-10 is positively associated with IL-6 and hs-CRP (Welsh et al., 2011[33]).

In conclusion, the results indicate a trend of reduced TC, LDL-C, and HDL-C and elevated TG levels along with inflammatory markers between day 1 and day 2 sample. However, corrections in the serum levels have been observed at day 7. Although the measurement of serum lipids is recommended after the admission of patients with ACS, serum lipid levels are measured in less than half of the patients. However, considering that phasic changes in serum lipid and lipoprotein levels occur after 24 hours of ACS, the findings of this study emphasize the need for assessment of the lipid profile of these patients to be made at admission, so as to identify patients at a higher potential risk. Exact knowledge regarding baseline serum lipids and lipoprotein levels as well as their varying characteristics can be used to guide selection of lipid lowering medication.

## Figures and Tables

**Table 1:**
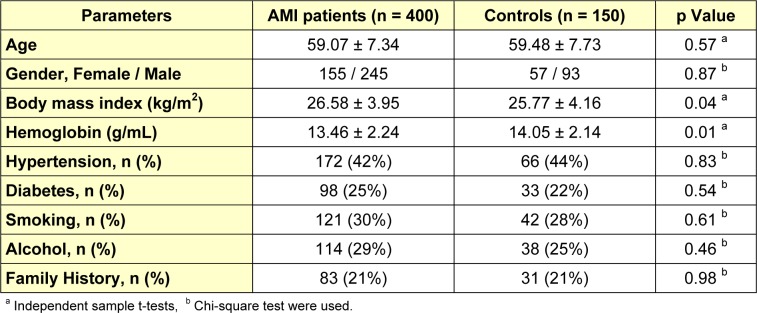
Clinical and demographic characteristics of AMI patients and controls

**Table 2:**
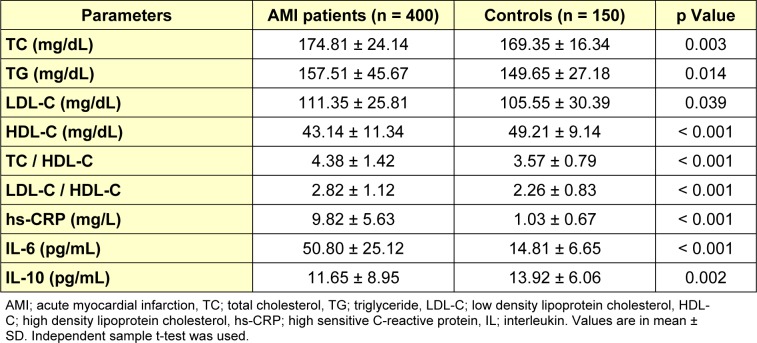
Mean levels of lipid profile and inflammatory markers in baseline serum sample of AMI patients and controls

**Table 3:**
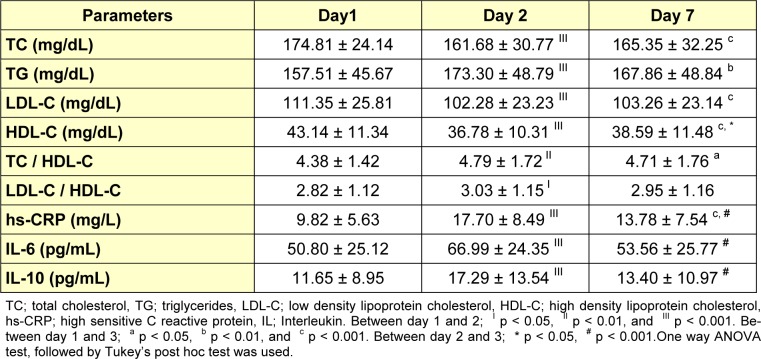
Comparison of serum lipid values and inflammatory markers between day 1, day 2 and day 7 post- AMI
